# Revealing the evolution of the tumor immune microenvironment in follicular lymphoma patients progressing within 24 months using single-cell imaging mass cytometry

**DOI:** 10.1186/s13045-022-01326-z

**Published:** 2022-08-22

**Authors:** Long Liu, Xingxing Yu, Zhifeng Li, Xiaohua He, Jie Zha, Zhijuan Lin, Yan Hong, Huijian Zheng, Qian Lai, Kaiyang Ding, Xian Jia, Guo Fu, Haifeng Yu, Haiyan Yang, Zhiming Li, Ken H. Young, Bing Xu

**Affiliations:** 1 Department of Hematology, The First Affiliated Hospital of Xiamen University and Institute of Hematology, School of Medicine, Xiamen University, Xiamen, 361003 People’s Republic of China; 2 Key Laboratory of Xiamen for Diagnosis and Treatment of Hematological Malignancy, Xiamen, 361003 People’s Republic of China; 3 Department of Medical Oncology, State Key Laboratory of Oncology in South China, Collaborative Innovation Center for Cancer Medicine, Sun Yat-sen University Cancer Center, 651 Dongfeng Road East, Guangzhou, People’s Republic of China; 4 Department of Hematology, The First Affiliated Hospital of USTC Anhui Provincial Hospital, Hefei, 230001 People’s Republic of China; 5 State Key Laboratory of Cellular Stress Biology, Innovation Center for Cell Signaling Network, School of Life Sciences, Xiamen University, Xiamen, 361005 People’s Republic of China; 6 Department of Lymphoma, Cancer Hospital of the University of Chinese Academy of Sciences, Zhejiang Cancer Hospital, Hangzhou, 310012 People’s Republic of China; 7 Department of Lymphoma, Institute of Cancer, and Basic Medicine (IBMC), Chinese Academy of Sciences, Hangzhou, 310012 People’s Republic of China; 8 Hematopathology Division, Department of Pathology, Duke University Medical Center, Durham, NC 27710 USA; 9 Duke University Cancer Institute, Durham, NC USA

**Keywords:** FL, TIME, POD, Immune signature, T cells

## Abstract

**Background:**

Patients with follicular lymphoma (FL) who experience disease progression within 24 months (POD24) have inferior outcomes. The tumor immune microenvironment (TIME) plays a crucial role in pathogenesis and progression of follicular lymphoma (FL). However, TIME evolution during progression of disease within 24 months (POD24) is elusive.

**Methods:**

Spatially resolved and single-cell image mass cytometry with a panel of 36 metal-tagged antibodies was used to quantitatively analyze the TIME structure in 13 paired FLs at diagnosis and POD24.

**Results:**

Follicles and peri-follicular regions were well dissected in structure. Peri-follicular regions represented a barrier for immune infiltration into the follicles. More FL-cells in the peri-follicular regions suffered CD8^+^T cells attacks under simultaneous protection of regulatory T cells (Tregs) and/or macrophages compared with that in the follicles irrespective of POD24. During POD24, increased CD163^−^ macrophages with PD-1 ligand upregulation and decreased CD8^+^T cells with upregulated LAG-3 expression around FL-cells were observed in the follicles. Spatial analyses demonstrated that FL-cells interacted more intimately with macrophages than with Tregs and less with cytotoxic T cells in both peri-follicular regions and follicles during POD24. In comparison, macrophages also cooperated more frequently with Tregs to simultaneously hijack FL-cells, creating an enhanced immunosuppressive environment in both peri-follicular and follicular regions during POD24.

**Conclusions:**

Peri-follicular regions function as a barrier by recruiting both CD8^+^T cells and immunosuppressive cells, protecting follicular FL-cells from immune attack at diagnosis or POD24. FL-cells reside in a more immune-compromised microenvironment and evade immune cell attacks during POD24. Novel immunotherapeutic approaches harnessing LAG-3, macrophages, and Tregs will be empowered to overcome poor outcomes in patients with FL POD24.

**Supplementary Information:**

The online version contains supplementary material available at 10.1186/s13045-022-01326-z.

## To editor

Patients with follicular lymphoma (FL) who experience disease progression within 24 months (POD24) have inferior outcomes [1, 2]. Current biological understanding of POD24 is mainly based on alterations acquired at the time of diagnosis of FL [3], including gene-mutation-based prognostic model POD24-PI [4] and TIME-based model BioFLIPI [5], but biological studies on POD24 stages are rare. TIME played an important role in the progression of FL [3, 5, 6]. However, due to the lack of technologies that enable comprehensive spatial analysis at single-cell resolution with high-throughput phenotypic information, the evolution of TIME over the course of POD24 remains elusive.

**Fig. 1:**
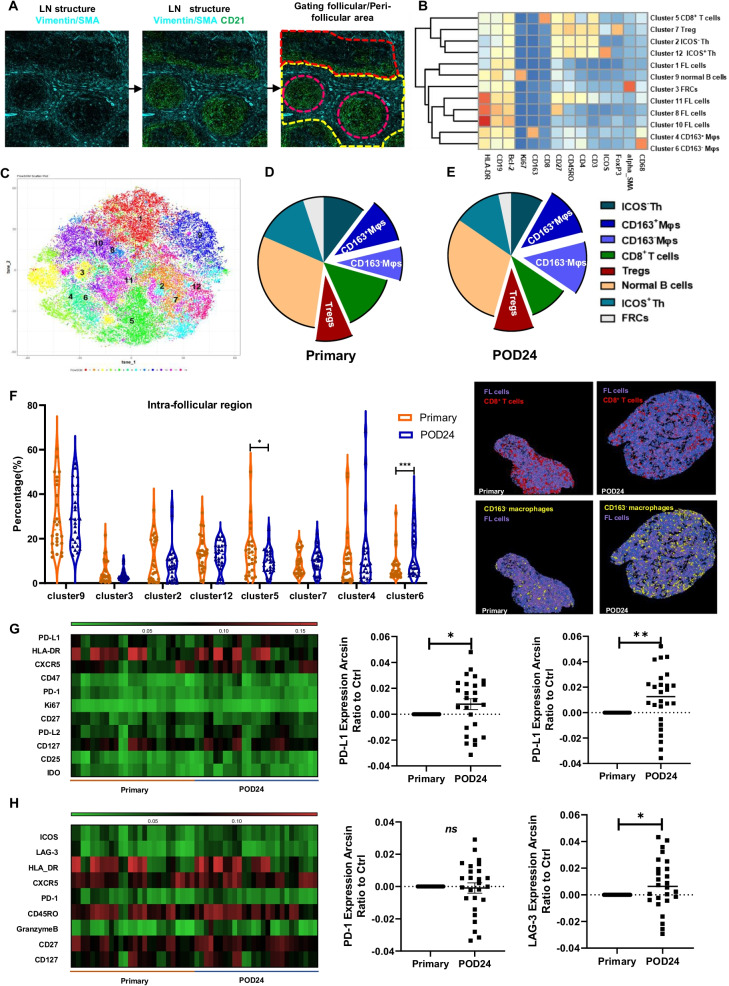
Evolution of immune components around FL-cells in the follicles during POD24. **A** The lymph node structure shows that areas between the follicular and peri-follicular boundaries were clearly displayed with SMA, vimentin, and CD21; **B**–**C** Cells were divided into 12 categories including four FL subsets (clusters 1, 8, 10, and 11), two subsets of CD4^+^ helper T cells (Th) classified by inducible T cell costimulator (ICOS) expression including ICOS^−^Th (cluster 2) and ICOS^+^Th (cluster 12), two types of tumor-associated Mφs (TAMs): CD163^+^Mφs (cluster 4) and CD163^−^Mφs (cluster 6), CD8^+^ T cells (cluster 5); normal B cells (cluster 9); and Tregs (cluster 7) and fibrotic reticular cells (FRCs, cluster 3) according to the indicated markers displayed by the heatmap (**B**) and t-SNE (**C**). **D**–**E** Pie charts of cellular components around FL-cells at diagnosis (**D**) and POD24 (**E**); **F** alterations in the frequency of immune cells surrounding FL-cells during POD24. **G** Heatmap of phenotypic alterations (left), and PD-L1/2 expression (right) in CD163^−^ macrophages during progression of disease within 24 months (POD24) in FL. **H** Heatmap of phenotypic alterations (left), and PD-1 and LAG-3 expression (right) in CD8^+^T cells during POD24 in FL. (*p < 0.05, **p < 0.01, ***p < 0.001, ordinate represent “Expression Arcsin Ratio to Ctrl,” which were calculated as follows: (Arcsin of POD24 – arcsin of primary)/Arcsin of primary

Imaging mass cytometry (IMC) is a novo technology which enables the simultaneous spatial and phenotypic evaluation of up to 50 biomarkers from tissue sections stained with metal-tagged antibodies at single-cell resolution [7, 8]. Therefore, we used IMC to analyze TIME evolution from diagnosis to POD24 using 36 metal-tagged antibodies in 13 FL patients. IMC clearly identified follicles, peri-follicular regions, and the boundary in-between according to CD21/SMA/vimentin expression (Fig. 1A). A total of 12 clusters were identified in the TIME (Fig. 1B, C). Not all cells in the TIME directly interacted with FL-cells due to spatial segmentation. Therefore, analysis of neighboring components around FL-cells could reflect more accurately the microenvironment that each FL-cell resides in than cellular components in the whole sections. Macrophages (Mφs) were most abundant immunosuppressive subpopulation, which were twice as numerous as regulatory T cells (Tregs) adjacent to FL-cells at diagnosis or at POD24 (Fig. 1D, E). Furthermore, among the components that surround FL-cells, the percentage of CD163^−^Mφs but not CD163^+^Mφs increased at POD24 (Fig. 1E, F). However, the frequencies of Tregs around FL-cells did not significantly change after POD24 (Fig. 1F). A decrease in CD8^+^ T cells around FL-cells after POD24 was observed (Fig. 1F). Further phenotypic analysis showed that CD163^−^Mφs around FL-cells displayed higher expression of PD-L1 and PD-L2 (Fig. 1G), while CD8^+^T cells neighboring FL-cells showed higher expression of lymphocyte activation gene 3 but not programmed cell death protein 1(PD-1) after POD24 (Fig. 1H). However, when comparing intra-follicular TIME at diagnosis in patients with and without POD24, immune components around FL-cells were similar between two groups (Additional file 1: Fig. S1A). Imbalanced evolution in CD163^−^Mφs and CD8^+^ T cells surrounding FL-cells might result in a more immunosuppressive TIME after POD24.

**Fig. 2:**
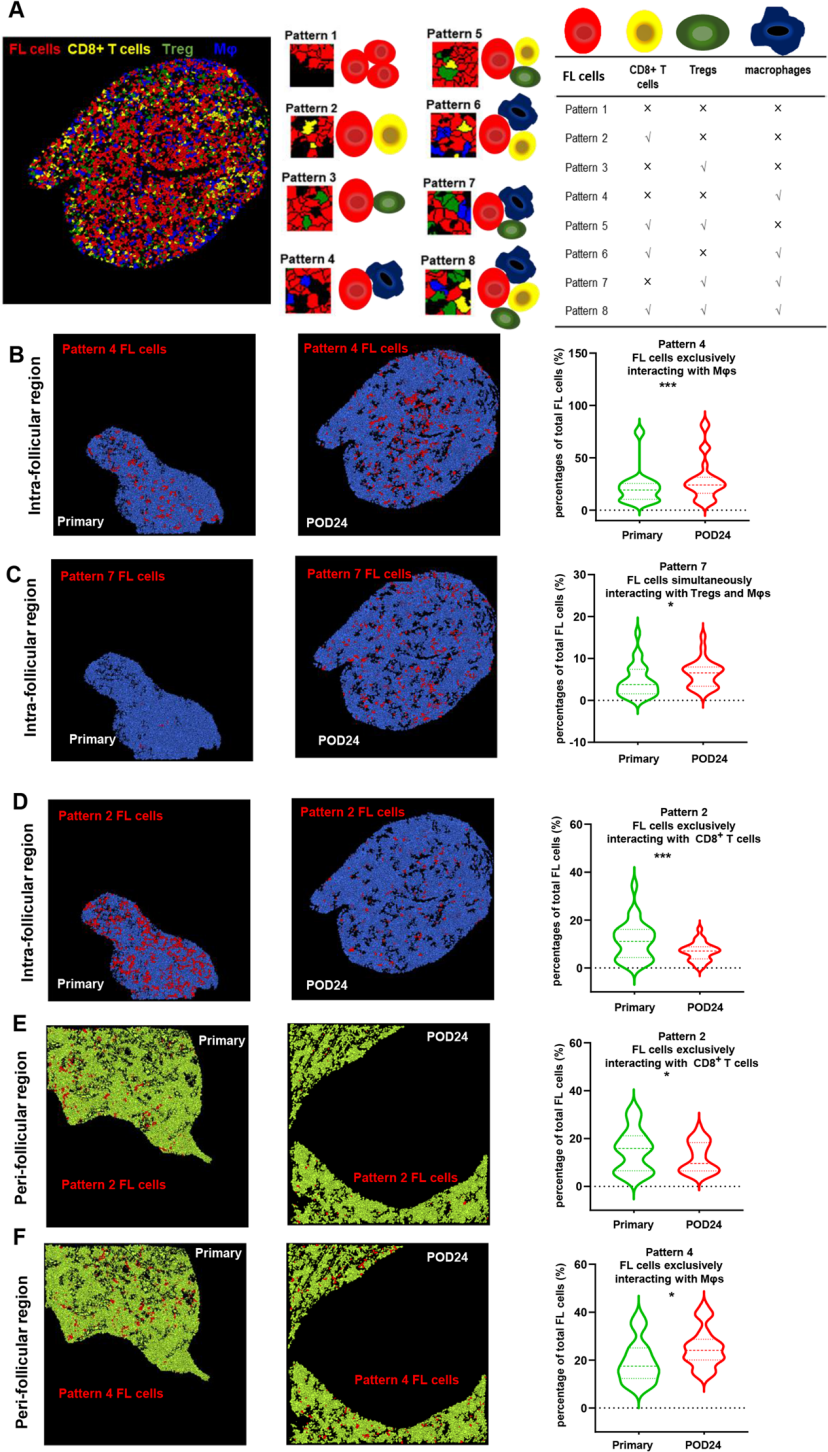
Evolution of FL and immune cell interactions during POD24 in FL (**A**), scheme of analysis of interactions between FL and three types of immune cells. Total eight interaction patterns are proposed in the table (right) according to the colocation of FL and immune cells. The fractions of FL-cells with different interactions accounting for total FL-cells were calculated. Higher percentages of FL-cells exclusively interacting with Mφs were shown in Pattern 4 (**B**), while those simultaneously interacting with Mφs and Tregs were shown in Pattern 7 (**C**) in the follicles during POD24 in FL. A significant decrease in FL-cells exclusively interacting with CD8^+^T cells was shown in Pattern 2 (**D**) after POD24. Additionally, in the peri-follicular regions, decreased FL-cells exclusively interacting with CD8^+^T cells were shown in Pattern 2 (**E**), whereas more FL-cells exclusively interacting with Mφs were shown in Pattern 4 (**F**) during POD24 in FL (*p < 0.05, **p < 0.01, ***p < 0.001)

Although the dynamic variations of FL-cells indicate the presence of immunosuppressive TIME after POD24, the evolution of spatial interactions between FL-cells and the immune cells is unknown. Eight interaction patterns were identified based on the colocalization of FL-cells with Mφs, Tregs, and CD8^+^T cells (Fig. 2A). Besides more exclusive interaction with Mφs in the follicular and peri-follicular regions (Pattern 4, Fig. 2B, F), more FL-cells simultaneously hijacked by Mφs and Tregs (Pattern 7) were observed in the follicles after POD24 (Fig. 2C). Furthermore, fewer FL-cells interacted with CD8^+^T cells (Pattern 2) without protection of immunosuppressive cells like Mφs or Tregs (Fig. 2D, E). Intriguingly, we found that peri-follicular regions represented a barrier for immune infiltration into the follicles. FL-cells in follicles were found to interact more with themselves (Additional file 1 Fig. S2A,B) and separated spatially from the attack by CD8^+^T cells than that in the peri-follicular regions. In contrast, FL-cells in the peri-follicular regions suffered more CD8^+^T cells attacks than in the follicles (Additional file 1: Fig. S2F). Notably, more FL-cells that interacted with CD8^+^T cells in the peri-follicular regions were simultaneously protected by Tregs and/or Mφs than in the follicles (Additional file 1: Fig. S2C). Even FL-cells distant from CD8^+^T cells in the outer areas were protected more frequently by the cooperation of Tregs and Mφs compared with that in the central regions (Additional file 1: Fig. S2C). Moreover, the barrier functions of peri-follicular regions were not changed after POD24 (Additional file 1: Fig. S2D, E).

In summary, IMC was applied to create a spatially preserved single-cell-resolution immune atlas from peri-follicular to follicular regions of FL, providing novel and significant insights of TIME evolution during POD24. An enhanced immunosuppressive TIME was emerged during POD24, characterized by increased TAMs and fewer CD8^+^T cells, higher expression of LAG-3 around FL-cells as well as more cooperation with Tregs and TAMs around the FL-cells. Consequently, new therapeutic approaches by modulating LAG-3, TAMs, or Tregs could potentially improve dismal survival in patients with POD24.

## Supplementary Information

**Additional file 1. **Study methods and supplementary figures  and tables.

## Data Availability

The original contributions presented in the study are included in the article/supplementary materials. The IMC data will be uploaded in the public repository “Zenodo”.

## Abbreviations

FDCs: Follicular dendritic cells
FL: Follicular lymphoma
FRCs: Fibrotic reticular cells
ICOS: Inducible T cell costimulator
IMC: Imaging mass cytometry
LAG-3: Lymphocyte activation gene 3
Mφs: Macrophages
PD-1: Programmed cell death protein 1
PD-L1: Programmed cell death protein ligand 1
PD-L2: Programmed cell death protein ligand 2
POD24: Progression of disease with 24 months
ROIs: Regions of interest
SMA: Smooth muscle alpha actin
TAMs: Tumor-associated macrophages
Tcons: Conventional CD4^+^ T cells
Tfhs: Follicular helper T cells
TIM-3: T cell immunoglobulin and mucin domain 3
TIME: Tumor immune microenvironment
Tregs: Regulatory T cells
