# Supplementary material for: Revealing the evolution of the tumor immune microenvironment in follicular lymphoma patients progressing within 24 months using single-cell imaging mass cytometry
Source: J Hematol Oncol. 2022 Aug 22;15:115. doi: 10.1186/s13045-022-01326-z (PMC9396877; doi:10.1186/s13045-022-01326-z)
Supplement: Supplementary file 1 — Additional file 1. Study methods and supplementary figures and tables. [file 13045_2022_1326_MOESM1_ESM.docx]

**Methods**

**Patient characteristics and sample preparation**

In total, 39 paraffin-embedded lymph node samples from FL patients, including 26 samples at diagnosis and 13 paired samples at POD24 from the First Affiliated Hospital of Xiamen University and Sun Yat-sen University Cancer Center from September 2001 to September 2018 were enrolled in this study. All patient detected positive Bcl-2 expression in FL-cells according to pathological information. The patient characteristics are summarized (supplementary table 1). Briefly, the median patient age was 57 years. Half of the patients were diagnosed with grades 1–2, and 42% (11/26) and 8% (2/26) were diagnosed with grades 3A and 3B disease, respectively. Most patients (24/26) received rituximab-based immunochemotherapy, except for two patients who underwent chemotherapy. After frontline therapy, PET/CT were used to evaluate the treatment response. 73% of patients achieved complete remission (CR), whereas 23% (6/26) achieved partial remission, and one patient remained stable. For patients with POD24, the median time to progression was 562 days. In total, 38% (5/13) failed to achieve CR after first-line therapy.

All tissue sections were produced to a thickness of 5 μm. Regions of interest (ROIs) covering the follicular and interfollicular areas were selected according to the locations of consecutive sections stained with hematoxylin-eosin. For patients with disease that transformed into diffuse large B-cell lymphoma, ROIs covering malignant follicles were selected according to CD21 and Bcl-2 expression in the germinal center. All samples were obtained after the patients provided informed consent. IRB approval for this study was granted by the Institutional Human Ethics Review Committee of the First Affiliated Hospital of Xiamen University and Sun Yat-sen University Cancer Center, in accordance with the Declaration of Helsinki.

**Antibody conjugation and staining**

For antibodies without available conjugated metal in Fluidigm, conjugated metal and primary antibodies were constructed as described previously[1]. Following conjugation, the antibodies were diluted in 0.5% BSA and stored at 4°C for staining. Slide-tissue sections were baked at 67°C for 30 min. Tissue sections were deparaffinized with two washes of 100% fresh-xylene and then rehydrated with successive washes with ethanol 100% (2×), 95% (2×), 85% (1×), 70% (1×), and distilled water. The sections were then immersed in antigen retrieval buffer (tris-EDTA buffer, pH 9.0; Abcam, Cambridge, UK), incubated at 97°C for 30 min, and cooled to room temperature at room temperature. Slides were washed with distilled water (1×) and Dulbecco’s phosphate-buffered saline (1×) for 10 min each and then washed with distilled water for 8 min on an orbital shaker. Next, the tissue was blocked with blocking buffer for 45 min at 25–30°C (Superblock Blocking Buffer in PBS; Thermo Fisher Scientific, Waltham, MA, USA) in a humid chamber. After blocking, the antibody mix was then applied with the dilutions indicated in supplementary table 2 and incubated overnight at 4 °C in a humid chamber. After overnight incubation, the slides were washed on an orbital shaker for 5 min in wash buffer. Thereafter, tissue sections were successively washed with 0.2% triton X-100 (2×) and D-PBS (2×) on an orbital shaker. Subsequently, slides were incubated with intercalator-Ir diluted 1:400 in D-PBS for 30 min at room temperature. After incubation, the sections were washed with distilled water for 5 min. Finally, the slides were dried under airflow for 5 min and stored at 4°C until ablation.

**IMC acquisition**

The Hyperion mass cytometry system (Fluidigm Corporation, South San Francisco, CA, USA) was autotuned using a 3-element tuning slide according to the tuning protocol provided by the manufacturer. As an extra threshold for successful tuning, a detection of at least 1,500 mean duals of 175 Lu was used. ROIs were selected based on consecutive tissue sections stained with hematoxylin and eosin. ROIs of 1,000 × 1,000 µm were ablated and acquired at 200 Hz for approximately 2 h. Data were exported as MCD ﬁles and visualized using the Fluidigm MCD^TM^ viewer. To better separate the antibody signal and noise, each marker was visually inspected, and a minimum signal threshold of one or two dual counts was set in the Fluidigm MCD^TM^ viewer.

**Cell segmentation**

For cell segmentation of non-immune cells, we employed DNA-Iridium staining as nuclear and cell membrane markers to define primary and secondary objects. CellProfiler was subsequently used to define the cell masks and quantify marker expression. To define cell borders, nuclei were first identified as primary objects based on ilastik probability maps and expanded through the cytoplasm compartment until either a neighboring cell or the background compartment was reached. Next, cell masks were generated to identify single cells and used to extract single-cell information (marker abundance, spatial, and neighborhood data) from the original images. Finally, the results were exported as .tiff for analysis.

**Image visualization and phenotypic analysis**

Each sample and mask were imported into HistoCAT version 1.76[2], and the intra- and inter-follicle regions were gated according to CD19 and SMA expression. After gating, each lymph node sample that was noted to morphologically span both the outer and inner stripes was exported from HistoCAT as a csv file, including the cell ID, signal strength of markers, and coordinates of each cell. Data were analyzed and graphed using R and HistoCAT software. the R implementation of FlowSOM (https://github.com/SofieVG/FlowSOM) was used to define the cell type. In particular, an R implementation of tSNE (https://cran.r-project.org/web/packages/tsne) was used to generate tSNE maps. Unless otherwise stated, the raw IMC counts were 99th-percentile-normalized and scaled from 0 to 1 (scaled counts) [3]. The comparisons were perfomed using Expression Arcsin Ratio to Ctrl”, which were calculated as follows: (Arcsin of pod24 – arcsin of primary)/Arcsin of primary.

**Neighborhood and cell interaction analysis**

Neighborhood analysis was performed using R and HistoCAT software. Custom scripts were used to import the cell type into HistoCAT. An expansion of four pixels from the center of cells was used to detect the cell neighbors[2]. Interaction pattern analysis for Tregs, Mφs, CD8^+^ T cells, and FL cells was performed as follows. A unique ID was assigned to each identified cell. The IDs of all cells surrounding a cell could be extracted in a csv file via HistoCAT. Based on the cellular phenotypes, cells were classified into different cell types including Tregs, Mφs, CD8^+^ T cells, and FL cells. We analyzed the interactions of each cell type with the other three cell types and further categorized the cells into eight groups according to the interaction patterns of four general interaction patterns as follows: simultaneous interaction with other three cell types, interaction with any two of three other cell types, interaction with only one of three other cell types, and no interactions with any other cell types (as shown in Figure 2A). The number of cells in each pattern could be calculated according to the cell IDs. All other parameters and methods were chosen as suggested in the original publication[2].

**Statistical analysis**

Statistical tests were performed using R and GraphPad Prism. The statistical parameters are reported in the figure legends. For pairwise group comparisons, we used a non-parametric paired statistical test when analyzing data before and after POD24 as well as data from follicular and peri-follicular regions. Overall, the *p*-value significance threshold was set at 0.05.

**References**

1. Han G, Spitzer MH, Bendall SC, Fantl WJ, Nolan GP. Metal-isotope-tagged monoclonal antibodies for high-dimensional mass cytometry. Nat Protoc. 2018;13(10):2121-48.

2. Schapiro D, Jackson HW, Raghuraman S, Fischer JR, Zanotelli VRT, Schulz D, et al. histoCAT: analysis of cell phenotypes and interactions in multiplex image cytometry data. Nature methods. 2017;14(9):873-6.

3. Levine JH, Simonds EF, Bendall SC, Davis KL, Amir el AD, Tadmor MD, et al. Data-Driven Phenotypic Dissection of AML Reveals Progenitor-like Cells that Correlate with Prognosis. Cell. 2015;162(1):184-97.

| **Antibody** | **Lable** | **Clone** | **CAT-NO** | **Company** | **dilution** |
| --- | --- | --- | --- | --- | --- |
| **T-bet** | **145Nd** | **D6N8B** | **3145015D** | **Fluidigm** | **100** |
| **PD-L1** | **150Nd** | **E1L3N** | **3150031D** | **Fluidigm** | **100** |
| **CD45** | **152Sm** | **CD45-2B11** | **3152016D** | **Fluidigm** | **100** |
| **CD11c** | **154Sm** | **Polyclonal** | **3154025D** | **Fluidigm** | **100** |
| **E-Cadherin** | **158Gd** | **24E10** | **3158029D** | **Fluidigm** | **100** |
| **CD56** | **161Dy** | **EPR2566** | **ab214436** | **Abcam** | **100** |
| **CD47** | **164Dy** | **CD47/2937** | **ab260480** | **Abcam** | **100** |
| **PD-1** | **165Ho** | **EPR4877(2)** | **3165039D** | **Fluidigm** | **100** |
| **Bcl-6** | **169Tm** | **K112-91** | **561520** | **BD** | **100** |
| **PD-L2** | **172Yb** | **D7U8C** | **3172031D** | **Fluidigm** | **100** |
| **CD127** | **173Yb** | **EPR2955(2)** | **ab240225** | **Abcam** | **100** |
| **CD3ε** | **170Er** | **Polyclonal** | **3170019D** | **Fluidigm** | **200** |
| **SMA** | **141Pr** | **1A4** | **3141017D** | **Fluidigm** | **200** |
| **CD19** | **142Nd** | **6OMP31** | **3142014D** | **Fluidigm** | **200** |
| **Vimentin** | **143Nd** | **D21H3** | **3143027D** | **Fluidigm** | **200** |
| **CD14** | **144Nd** | **EPR3653** | **3144025D** | **Fluidigm** | **200** |
| **BCL-2** | **146Nd** | **EPR17509** | **3146019D** | **Fluidigm** | **200** |
| **CD163** | **147Sm** | **EDHu-1** | **3147021D** | **Fluidigm** | **200** |
| **ICOS** | **148Nd** | **D1K2T** | **3148021D** | **Fluidigm** | **200** |
| **CD35** | **149Sm** | **E11** | **ab25** | **Abcam** | **200** |
| **CD31** | **151Eu** | **EPR3094** | **3151025D** | **Fluidigm** | **200** |
| **LAG-3** | **153Eu** | **D2G40** | **3153028D** | **Fluidigm** | **200** |
| **FoxP3** | **155Gd** | **236A/E7** | **3155016D** | **Fluidigm** | **200** |
| **CD4** | **156Gd** | **EPR6855** | **3156033D** | **Fluidigm** | **200** |
| **CD68** | **159Tb** | **KP1** | **3159035D** | **Fluidigm** | **200** |
| **CD8a** | **162Dy** | **C8/144B** | **3162034D** | **Fluidigm** | **200** |
| **CXCR5** | **163Dy** | **51505** | **MAB190-100** | **R&D** | **200** |
| **CD45RO** | **166Er** | **UCH-L1** | **ab23** | **Abcam** | **200** |
| **Granzyme B** | **167Er** | **EPR20129-217** | **3167021D** | **Fluidigm** | **200** |
| **Ki-67** | **168Er** | **B56** | **3168022D** | **Fluidigm** | **200** |
| **CD27** | **171Yb** | **EPR8569** | **3171024D** | **Fluidigm** | **200** |
| **CD21** | **174Yb** | **SP186** | **ab240987** | **Abcam** | **200** |
| **CD25** | **175Lu** | **EPR6452** | **3175036D** | **Fluidigm** | **200** |
| **IDO** | **176Yb** | **D5J4E** | **ab238788** | **Abcam** | **200** |
| **HLA-DR** | **160Gd** | **HLA-Pan/2967R** | **ab259258** | **Abcam** | **400** |
| **DNA1/2** | **191Ir** | **Cell-ID™ Intercalator-Ir—125 µM** | **201192B** | **Fluidigm** | **400** |

**Additional file 1: Table S1**

| **Additional file 1: Table S2** | | | | | | | | |
| --- | --- | --- | --- | --- | --- | --- | --- | --- |
| **Number** | **Gender** | **Age(years)**  **at diagnosis** | **Grade (1-2=0; 3A=1; 3B=2)** | **Lugano stage** | **FLIPI-2** | **Induction Regimens** | **Response** | **POD24**  **Yes=1,No=0** |
| A03 | female | 59 | 2 | IVA | 3 | R-CHOP | SD | 1 |
| A04 | female | 64 | 0 | IIA | 1 | R-CHOP | CR | 1 |
| A05 | female | 64 | 2 | IA | 3 | R-CHOP | PR | 1 |
| A06 | male | 51 | 1 | IIIA | 3 | RCHOP | PR | 1 |
| A07 | female | 43 | 1 | ⅢA | 3 | RCHOP | CR | 1 |
| A09 | female | 56 | 1 | III A | 2 | R-CHOP+BR | CR | 1 |
| A10 | male | 60 | 0 | IIIA | 3 | BR | PR | 1 |
| A11 | male | 50 | 0 | IVB | 0 | RCHOP+ISRT | CR | 1 |
| A12 | male | 55 | 1 | IIIA | 2 | R-CHOP | CR | 1 |
| A13 | female | 31 | 1 | IIIA | 2 | R-CHOP | PR | 1 |
| A14 | male | 48 | 0 | III A | 2 | R-CHOP | CR | 1 |
| A15 | male | 45 | 0 | IV B | 2 | FMD | CR | 1 |
| A16 | male | 60 | 0 | IV A | 3 | RCHOP | CR | 1 |
| C01 | male | 53 | 0 | IIIA | 1 | RCHOP | CR | 1 |
| C02 | female | 54 | 1 | IIIA | 0 | RCHOP | CR | 0 |
| C03 | male | 80 | 1 | IV B | 3 | RCHOP | CR | 0 |
| C04 | male | 52 | 0 | IIIA | 3 | RCHOP | CR | 0 |
| C05 | female | 49 | 0 | IIIB | 1 | R-CHOP | CR | 0 |
| C07 | male | 51 | 1 | IIA | 0 | CHOP+ISRT | CR | 0 |
| C08 | female | 62 | 0 | IVA | 3 | R-CHOP | CR | 0 |
| C09 | male | 52 | 1 | IIIB | 2 | R-CHOP | CR | 0 |
| C11 | male | 60 | 0 | ⅢA | 1 | BR | CR | 0 |
| C15 | male | 51 | 0 | IIIA | 0 | R-FC+ISRT | PR | 0 |
| C16 | female | 47 | 0 | IIIA | 0 | RCHOP+ISRT | CR | 0 |
| C17 | male | 77 | 1 | IIIB | 1 | RCHOP+ISRT | CR | 0 |
| C18 | male | 35 | 1 | IA | 0 | RCHOP+ISRT | PR | 0 |
| Abbreviations: BR: Bendamustine+ Rituximab; CR: complete remission; FMD: Fludarabine+ Mitoxantrone+ Dexamethasone; ISRT: involved sites irradiation therapy; PR: partial remission; R-CHOP: Rituximab+ Cyclophosphamide+ Doxorubicin+ Vincristine+ Prednisone; R-FC: Rituximab+ Fludarabine+ Cyclophosphamide. | | | | | | | | |

**A**

**Additional file 1: Figure S1. Comparison of immune components around FL-cells between patients with and without** **progression of disease within 24 months (POD24)**

1. Immune components of follicular regions in patients with POD24 and patients without POD24(non-POD24).

**B**

**C**

**D**

**E**

**F**


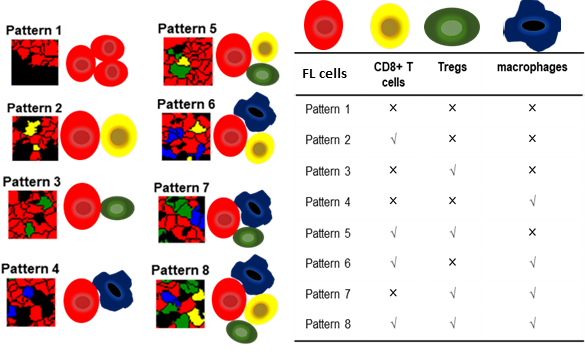


**A**


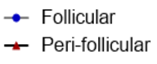

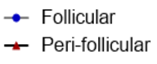

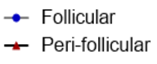


**Additional file 1: Figure S2. Evolution of follicular lymphoma (FL) and immune cell interactions during progression of disease within 24 months (POD24) in FL**

(A) Scheme of analysis of interactions between FL and three types of immune cells. In total, eight interaction patterns are listed in the table (right) according to the co-location of FL and immune cells. The fractions of FL cells with different interactions accounting for total FL cells were calculated. (B-C) The alterations of percentages of FL-cells interacting with mono-type of cells as shown in pattern1-4 (B) and simultaneously multiple cell types as shown in pattern 5-8 (C) from the outer to central regions at diagnosis of FL. (D-E) The alterations of percentages of FL-cells interacting with mono-type of cells as shown in pattern1-4 (D) and simultaneously multiple cell types as shown in pattern 5-8 (E) from the outer to central regions at POD24 of FL.(F): The alterations of percentages of FL-cells interacting with CD8^+^ T cells regardless of simultaneous interacting with other cell type from the outer to central regions.
